# Trichloronitromethane: MAK Value Documentation, addendum – Translation of the German version from 2023

**DOI:** 10.34865/mb7606e10_2ad

**Published:** 2025-06-30

**Authors:** Andrea Hartwig, MAK Commission

**Affiliations:** 1 Institute of Applied BiosciencesDepartment of Food Chemistry and ToxicologyKarlsruhe Institute of Technology (KIT) Adenauerring 20a, Building 50.41 76131 Karlsruhe Germany; 2 Permanent Senate Commission for the Investigation of Health Hazards of Chemical Compounds in the Work AreaDeutsche Forschungsgemeinschaft, Kennedyallee 40, 53175 Bonn, Germany. Further information: Permanent Senate Commission for the Investigation of Health Hazards of Chemical Compounds in the Work Area | DFG

**Keywords:** Trichlornitromethan, Pestizid, Bodenbegasung, Toxizität, Bewertung, trichloronitromethane, pesticide, soil fumigant, toxicity, evaluation

## Abstract

Trichloronitromethane (chloropicrin) [76-06-2] is used as a soil fumigant. It is no longer approved in the European Union. The previous MAK value documentation and addendum do not reflect the current data situation of the substance. The MAK Commission decided that a new evaluation is not of high priority. The MAK value and the other classifications are therefore suspended and the substance is listed in the Section II c of the List of MAK and BAT Values for substances no longer evaluated.

**Table:** 

**MAK value**	**see Section II c of the List of MAK and BAT Values**
**Peak limitation**	**–**
	
**Absorption through the skin**	**–**
**Sensitization**	**–**
**Carcinogenicity**	**–**
**Prenatal toxicity**	**–**
**Germ cell mutagenicity**	**–**
	
**BAT value**	**–**
	
Synonyms	chloropicrin nitrochloroform nitrotrichloromethane
Chemical name (IUPAC)	trichloro(nitro)methane
CAS number	76-06-2
Molecular formula	CCl_3_NO_2_
Molar mass	164.37 g/mol
Melting point	–64 °C (IFA 2022)
Boiling point	111.9 °C (IFA 2022)
Density at 20 °C	1.6448 g/cm^3^ (IFA 2022)
Vapour pressure at 50 °C	32 hPa (IFA 2022)
log K_OW_	2.09 (IFA 2022)
Solubility	2.27 g/l water (IFA 2022)
**1 ppm ≙ 6.82 mg/m^3^**	**1 mg/m^3^ ≙ 0.147 ml/m^3^ (ppm)**

This addendum was prepared because the published evaluation no longer reflects the data currently available for the MAK value and for the designation and classification of the substance.

Trichloronitromethane (chloropicrin) is used as a soil fumigant. In agriculture, the substance is injected into the soil prior to planting to control fungi, nematodes and diseases. It was used as a chemical weapon in World War I. Trichloroni﻿tromethane is severely irritating to the conjunctiva and cornea. The mechanism of action is presumably based on the reaction with biological thiols, which results in rapid dechlorination (Greim 2000, available in German only; Sparks et al. 1997). The MAK value of 0.1 ml/m^3^ (0.68 mg/m^3^) was determined to be below the odour and irritation threshold as early as 1961. In 2000, the substance was classified in Peak Limitation Category I with an excursion factor of 1 (Greim 2000; Henschler 1974, available in German only).

In the Pesticides Database of the European Union, the status of trichloronitromethane is listed as “not approved” (Euro﻿pean Commission 2023). An application to approve trichloronitromethane according to Regulation (EC) No 1107/2009 concerning the placing of plant protection products on the market (European Parliament and European Council 2009) was withdrawn in January 2022 because it was not possible to dispel the concerns raised by the authority. Therefore, trichloronitromethane is not approved as an active substance according to Regulation (EC) No 1107/2009 (European Commission 2022 b). Trichloronitromethane is on the list of chemicals in Annex I Parts 1 and 2 of the PIC (Prior Informed Consent) Regulation (EU) No 649/2012 (European Commission 2022 a). Exporters are thus required to submit notification of their intention to export this substance and receive explicit consent from the importing country prior to export.

In the Federal Republic of Germany, trichloronitromethane was approved for use from 1971 to 1976. The substance has been banned since 1980 (BVL 2010).

The previous evaluation does not reflect the currently available data. However, a re-evaluation of the substance is not a priority. Therefore, the MAK value and the peak limitation have been withdrawn and trichloronitromethane has been allocated to Section II c of the List of MAK and BAT Values (DFG 2022). This section lists substances for which the previous MAK values, designations and classifications have been withdrawn and which are no longer being reviewed at present.
